# PD-L1 in Oral Cavity Cancers—Audit for Tertiary Care Center in India

**DOI:** 10.1007/s13193-025-02195-x

**Published:** 2025-04-26

**Authors:** Zoya Peelay, Vijay Patil, Neha Mittal, Vanita Noronha, Nandini Menon, Ajaykumar Singh, Minit Shah, Shruti Pathak, Kumar Prabhash

**Affiliations:** 1 Medical Oncology, Tata Memorial Hospital, Homi Bhabha National Institute, Mumbai, India; 2 Pathology, Tata Memorial Hospital, Homi Bhabha National Institute, Mumbai, India

**Keywords:** Programmed death-ligand 1, Head and neck cancer, Tumor proportion score, Oral cavity cancer

## Abstract

There is no data on the expression of PD-L1 in oral cavity cancers from the Indian population. Hence, this audit was done to estimate the incidence of PD-L1 expression in oral cavity cancers and detect factors affecting the same. Data of 340 cases of oral cavity cancer who were advised for PD-L1 gene expression testing were collected from the head and neck OPD of Tata Memorial Hospital from the year 2018 to 2023. These cases were evaluated for demographic details, i.e., age and gender, and also for factors such as performance status (PS) as per the Eastern Cooperative Oncology Group (ECOG) scale, subsite of oral tumor, histopathology, and grade. Descriptive statistics were used for analysis. Factors affecting PD-L1 gene expression were sorted using ordinal logistic regression analysis. In total, 340 patients were evaluated with a median age of 48 years (range, 17–79; interquartile range, 40–55), and PD-L1 expression was divided as scores and was observed that Tumor Proportion Score (TPS) 0% was seen in 34 patients (10.0%), TPS 1–5% was seen in 70 patients (20.6%), TPS 6–10% was seen in 29 patients (8.5%), TPS 11–20% was seen in 29 patients (8.5%), TPS 21–30% was seen in 33 patients (9.7%), TPS 31–50% was seen in 44 patients (12.9%), TPS 51–75% was seen in 47 patients (13.8%), and TPS > 75% was seen in 54 patients (15.9%). Univariate analysis was run to determine the distribution of TPS scores within each variable under age, gender, sample collection site, differentiation of tumor, and subsite of tumor. This is one of the first studies evaluating data on the expression of PDL-1 in oral cavity cancers in the Indian population and the factors affecting it. The data provides novel insights into many factors potentially affecting the expression of PDL-1 in oral cavity cancers and in the future, can be of help in developing treatment plans with various immunotherapies.

## Background

Head and neck (HN) cancers constitute one of the most prevalent cancer groups in India, with oral cavity cancers accounting for a significant proportion due to the widespread habit of tobacco and betel nut chewing with an incidence of 17.02%, with lip and oral cavity cancers making up 10.3%, as per the data published by the global cancer observatory in the year 2020 [1, 2]. A wide majority of them present in the locally advanced stage [3] and most require palliative systemic therapy at some point. Immunotherapy is a part of standard palliative treatment in recurrent or metastatic head and neck cancers [4]. Nivolumab in comparison with chemotherapy (docetaxel or methotrexate)/targeted therapy (cetuximab) in platinum-refractory head and neck cancers has shown significant improvement in overall survival [5, 6]. The estimated 1-year survival rate with nivolumab was approximately 19% higher in comparison with the other therapies (36.0% vs. 16.6%; *p*-value = 0.01) [7]. A similar improvement was shown by pembrolizumab, where the median overall survival in the intention-to-treat population was 8.4 months (95% CI 6.4–9.4) with pembrolizumab and 6.9 months (5.9–8.0) with the standard of care (docetaxel or methotrexate or cetuximab) with a nominal *p*-value of 0.016 (8). Hence, either nivolumab or pembrolizumab is recommended as the standard treatment option in platinum-refractory head and neck cancers by international guidelines [4]. In the first-line setting, pembrolizumab plus platinum and 5-fluorouracil is an appropriate first-line treatment as per KEYNOTE-048 for recurrent or metastatic head and neck squamous cell carcinoma (HNSCC) with programmed cell death-ligand 1 (PD-L1) combined positive score (CPS) 1–20% and pembrolizumab monotherapy is an appropriate first-line treatment for recurrent or metastatic oral cavity PD-L1 CPS ≥ 20% and has received FDA approval for the same [8, 9].

Biomarker-based treatment in immunotherapy is beneficial in oral cancers. Ferris et al. suggested that higher PD-L1 expression is associated with higher overall survival in head and neck cancer patients. Patients with > 50% PD-L1 expression have a higher overall survival (OS) as compared to patients with lower PD-L1 expression [7]. Similar trends with PD-L1 expression were also seen in KEYNOTE-048. Pembrolizumab with chemotherapy improved overall survival versus cetuximab with chemotherapy in the total population (13.0 months vs. 10.7 months, *p* = 0.003) at the second interim analysis and in the CPS of 20 or more population (14.7 vs. 11.0, *p*-value =  < 0.001) and CPS of 1 or more population (13.6 vs. 10.4, *p* < 0.001) at the final analysis. Hence, the magnitude of benefit in overall survival was based on PD-L1 expression measured whether as Tumor Proportion Score (TPS) or CPS. As per our knowledge, there is no data about the incidence of PD-L1 expression in oral cavity cancers in India. Given the distinct pathophysiology of these cancers—primarily tobacco-related rather than HPV-driven—our study aimed to estimate PD-L1 expression in oral cavity cancers and analyze associated clinicopathological factors.

## Methods

The head and neck medical oncology OPD of Tata Memorial Hospital, Mumbai, maintains a database of all the oral cavity cancer patients receiving systemic therapy. The study was approved by the Institutional Ethics Committee. Patients who were advised for PD-L1 testing were identified from the database from the year 2018 to 2023. A total of 340 cases were identified for which PD-L1 testing was performed. Tumor Proportion Score (TPS) scoring for PD-L1 expression was performed using VENTANA PD-L1 assay. The antibody used in this procedure was the SP263 clone. PD-L1 status was determined by the percentage of tumor cells with any membrane staining above the background. The TPS value was represented in percentage.

### Data Collection

Demographic variables such as age, gender, performance status (PS) as per the Eastern Cooperative Oncology Group (ECOG) scale, oral cavity subsite of tumor, histopathology, and grading were collected from the palliative chemotherapy database and hospital electronic medical records (EMR). The status of the patient and date of death was collected from the electronic medical records and/or palliative chemotherapy database.

### Statistical Analysis

Descriptive statistics was performed. The ordinal and nominal variables were expressed in terms of percentages while continuous variables were expressed in terms of the median with interquartile range (IQR). PD-L1 expression was divided as scores TPS 0%, TPS 1–5%, TPS 6–10%, TPS 11–20%, TPS 21–30%, TPS 31–50%, TPS 51–75%, and TPS > 75%. The PD-L1 expression was tabulated for the entire population. Factors affecting PD-L1 expression were sought using ordinal logistic regression analysis. The dependent variable was PD-L1 expression categorized in ascending TPS as stated above and the independent variables were age (elderly versus non-elderly), gender (male versus female), sample collection site (biopsy versus resection), differentiation of tumor, and oral cavity subsite of the tumor. A *p*-value of 0.05 or below was considered significant.

## Results

### Baseline Characteristics

The baseline characteristics are shown in Table 1.

**Table 1:** Baseline characteristics of the cohort. *ECOG-PS*, Eastern Cooperative Oncology Group—Performance Status; *GBS*, gingivobuccal sulcus

Variable	Value
**Age—no. (%)**	
Median	48
Interquartile range	40–55
**Gender—no. (%)**	
Male	306 (90.0)
Female	34 (10.0)
**ECOG PS—no. (%)**	
0–1	253 (74.4)
≥ 2	71 (20.9)
Data not available	16 (4.7)
**Site—no. (%)**	
Tongue	89 (26.2)
Buccal mucosa	146 (42.9)
Floor of mouth	4 (1.2)
Palate	19 (5.6)
GBS	15 (4.4)
Alveolus	35 (10.3)
Retromolar trigone	24 (7.1)
Data missing	8 (2.4)
**Histology—no. (%)**	
Squamous cell carcinoma	332 (97.6)
Sarcomatoid	8 (2.4)
**Grade—no. (%)**	
Well differentiated	12 (3.5)
Moderately differentiated	200 (58.8)
Poorly differentiated	66 (19.4)
Sarcomatoid	8 (2.4)
Grading not available	54 (15.9)

### PD-L1 Expression

PD-L1 expression in the whole cohort is shown in Table 2. The factors impacting PD-L1 expression on univariate analysis are shown in Table 3. PD-L1 testing was performed on biopsy specimens in 297 (87.4%) patients. Resection was done on 43 (12.6%) specimens.

**Table 2:** PD-L1 expression in cohort. *TPS*, Tumor Proportion Score

Variable	Value
**TPS-(%)**	
Median	30
Interquartile range	5–60
**TPS category—no. (%)**	
TPS 0%	34 (10.0)
TPS 1–5%	70 (20.6)
TPS 6–10%	29 (8.5)
TPS 10–20%	29 (8.5)
TPS 20–30%	33 (9.7)
TPS 30–50%	44 (12.9)
TPS 50–75%	47 (13.8)
TPS > 75%	54 (15.9)

**Table 3:** Univariate analysis of factors affecting PD-L1 expression. *TPS*, Tumor Proportion Score; *PDSCC*, poorly differentiated squamous cell carcinoma; *MDSCC*, moderately differentiated squamous cell carcinoma; *WDSCC*, well-differentiated squamous cell carcinoma; *GBS*, gingivobuccal sulcus

**TPS → **		**0%**	**1–5%**	**6–10%**	**10–20%**	**20–30%**	**30–50%**	**50–75%**	** > 75%**	***p*-value**
**Variable↓**										
Age	Elderly(*n* = 53)	5(9.4)	12(22.6)	6(11.3)	9(17.0)	5(9.4)	4(7.5)	2(3.8)	10(18.9)	**0.074**
	Elderly(*n* = 287)	29(10.1)	58(20.5)	23(8.1)	20(7.1)	28(9.9)	40(14.1)	45(15.9)	44(15.5)	
Gender	Female(*n* = 34)	4(11.8)	6(17.6)	5(14.7)	2(5.9)	4(11.8)	1(2.9)	6(17.6)	6(17.6)	**0.480**
	Male(*n* = 306)	30(9.8)	64(20.9)	24(7.8)	27(8.8)	29(9.5)	43(14.1)	41(13.4)	48(15.7)	
Sample collection site	Biopsy(*n* = 297)	31(10.4)	59(19.9)	28(9.4)	24(8.1)	27(9.1)	39(13.1)	40(13.5)	49(16.5)	**0.577**
	Resection(*n* = 43)	3(7.0)	11(25.6)	1(2.3)	5(11.6)	6(14.0)	5(11.6)	7(16.3)	5(11.6)	
Tumor differentiation	PDSCC(*n* = 66)	5(7.6)	10(15.2)	4(6.1)	4(6.1)	2(3.0)	8(12.1)	13(19.7)	20(30.3)	** < 0.001**
	MDSCC(*n* = 200)	19(9.5)	40(20.0)	21(10.5)	21(10.5)	19(9.5)	29(14.5)	27(13.5)	24(12.0)	
	WDSCC(*n* = 12)	3(25.0)	3(25.0)	2(16.7)	1(8.3)	1(8.3)	1(8.3)	1(8.3)	0(0.0)	
	Sarcomatoid(*n* = 8)	0(0.0)	0(0.0)	0(0.0)	1(12.5)	0(0.0)	1(12.5)	0(0.0)	6(75.0)	
	Not mentioned(*n* = 54)	7(13.0)	17(31.5)	2(3.7)	2(3.7)	11(20.4)	5(9.3)	6(11.1)	4(7.4)	
	Tongue(*n* = 89)	4(4.5)	21(23.6)	6(6.7)	9(10.1)	11(12.4)	8(9.0)	13(14.6)	17(19.1)	** < 0.001**
	Buccal mucosa(*n* = 146)	17(11.6)	26(17.8)	16(11.0)	9(6.2)	10(6.8)	23(15.8)	23(15.8)	22(15.1)	
	Floor of mouth(*n* = 4)	0(0.0)	0(0.0)	0(0.0)	2(50.0)	0(0.0)	1(25.0)	0(0.0)	1(25.0)	
	Palate(*n* = 19)	1(5.3)	4(21.1)	0(0.0)	2(10.5)	4(21.2)	5(26.3)	1(5.3)	2(10.5)	
	GBS(*n* = 15)	0(0.0)	5(33.3)	2(13.3)	0(0.0)	3(20.0)	1(6.7)	2(13.3)	2(13.3)	
	Alveolus(*n* = 35)	4(11.4)	8(22.9)	2(5.7)	5(14.3)	2(5.7)	1(2.9)	7(20.0)	6(17.1)	
	Retromolar trigone(*n* = 24)	7(29.2)	5(20.8)	3(12.5)	1(4.2)	2(8.3)	3(12.5)	0(0.0)	3(12.5)	

## Discussion

This study is one of the first to evaluate PD-L1 expression in oral cavity cancers in the Indian population and the factors affecting its expression. The uniqueness of this study lies in its analysis of a large cohort of tobacco-related oral cancers, which differ significantly from HPV-associated cancers seen in the Western world.

Due to the habit of chewing tobacco and betel nuts, oral cancer is highly predominant in this region [10]. Due to the differences in sexual practices, the oropharynx cancer seen in India is not commonly associated with HPV [11, 12]. The rate of HPV-positive oropharyngeal cancer in accordance with a systematic review cumulatively is 28.85% in India [13]. The same in North America ranges between 65.4 and 72.2% [14]. HPV positivity influences PD-L1 expression and tumors with high HPV positivity tend to have high PD-L1 expression [15]. Hence, the importance of this data is that it gives insight into PD-L1 expression in unique oral cavity cancers with different pathophysiology compared to the Western world and are predominantly tobacco-related.

Overall, the PD-L1 expression of 1% or more in oral cavity tumors was seen in about 90% of the population. This is in comparison to the expression of PD-L1 in HNSCC in this population which is usually around 40–50%. In the Indian population, oral cancer is predominant as opposed to the pattern of cancer incidence in the Western population [10]. On univariate analysis, the distribution of TPS amongst the various factors is shown in Table 3. In our setting, PD-L1 expression greater than 1 and 50% are seen in patients 90.0% and 29.7%, respectively. Neoadjuvant pembrolizumab and nivolumab have shown excellent results when administered before surgery in phase 2 settings and phase 3 studies are ongoing [16–18]. In phase 2 studies, tumors with high PD-L1 expression derived additional benefits from the use of these agents [18]. On the basis of our data, it seems reasonable to try these checkpoint inhibitors on oral cancers, especially tobacco-related in our setting, where high PD-L1 positivity rate and expression are seen.

Our data suggests that the PD-L1 expression is high in poorly differentiated squamous cell carcinoma (PDSCC) compared to well-differentiated squamous cell carcinoma (WDSCC) with > 50% TPS as 50% in PDSCC, 25.5% in MDSCC, and just 8.3% in WDSCC. This data showed > 50% TPS in 75% of sarcomatoid histological subtype but the sample size was very small and other relevant data from extremity tumors has shown PD-L1 expression is low in sarcomas [19].

The data demonstrated a statistically significant association between the tumor site and PD-L1 expression (*p* < 0.001). For instance, tumors from the tongue and buccal mucosa had higher proportions of PD-L1 expression above the TPS > 50% threshold (19.1% and 15.1%, respectively). This association has been corroborated in other studies, such as Chow et al. (2020), which also found site-specific variations in PD-L1 expression [20]. These findings suggest that tumor microenvironmental factors, specific to the site, may influence PD-L1 expression, a topic that warrants further investigation.

The PD-L1 expression in biopsy and resected specimens did not show a statistically significant difference (*p* = 0.577). This is consistent with findings from other studies that have compared PD-L1 expression across different specimen types. For instance, studies by Bell et al. (2018) and Rimm et al. (2021) have shown similar PD-L1 expression levels between biopsies and resected specimens, suggesting that specimen type does not significantly impact the results of PD-L1 testing [21, 22]. However, further prospective studies with larger sample sizes are needed to confirm this finding.

The association of PD-L1 expression with tumor differentiation observed in this study aligns with findings from other cohorts. Poorly differentiated squamous cell carcinomas (PDSCC) exhibited the highest proportion of TPS > 50% scores (30.3%). Studies from Ferris et al. and others have similarly noted that poorly differentiated tumors tend to show higher PD-L1 expression, possibly due to increased immune evasion mechanisms [23].

Regarding age, gender, and specimen type, this study did not find significant differences in PD-L1 expression. This is consistent with data from previous studies, such as those by Kim et al. (2019), which also reported no statistically significant impact of these variables on PD-L1 expression [24]. Several factors were analyzed for their potential to alter PD-L1 expression. The statistical significance observed for tumor site and histological differentiation suggests these as key variables influencing PD-L1 levels. This observation aligns with prior studies that have linked tumor microenvironment characteristics, genetic mutations, and inflammation patterns to variations in PD-L1 expression. Further prospective studies with molecular analysis would be crucial to identify additional factors affecting PD-L1 expression in oral cavity cancers.

Our study has its own strengths and limitations. The strength is this is one of the first for such data from India and has a large sample size. The weakness is that for the patients included in this study, all PD-L1 testing was performed using the SP263 VENTANA platform. However, a comparison of this platform with the 22C3 PharmDx assay in head and neck cancer has been performed by Cerbelli et al. and the correlation between the two assays was reasonably good at 0.9 [25].

## Conclusion

PD-L1 expression of 1% or more was observed in 90.0% of oral cavity cancers in India, with 29.7% of patients exhibiting TPS > 50%. Notably, tumor site and differentiation were statistically significant factors influencing PD-L1 expression. This is one of the first studies providing real-world data on PD-L1 expression in Indian oral cancers, offering insights into unique, tobacco-related cancers distinct from HPV-associated cancers in the West.

The data provides novel insights into multiple factors potentially affecting the expression of PDL-1 in oral cavity cancers and can be of help in developing treatment plans with various immunotherapies in the future.

This study sets a benchmark for PD-L1 research in oral cavity cancers in India and underscores the potential role of immunotherapy in this population. Addressing the limitations and extending this work could help shape future biomarker-driven treatment strategies in Indian oncology practice. Further research is needed to explore additional biological and environmental factors influencing PD-L1 expression, with implications for the personalized use of immunotherapy in oral cavity cancers in India. These findings pave the way for personalized immunotherapy strategies, offering hope for improved outcomes in this distinct cohort.

## Data Availability

The individual de-identified data will be available on request to the corresponding author, until 5 years after publication. Requests beyond this timeframe will be considered on a case-by-case basis.

## References

[1] Globocan (2012) - Home [Internet]. [cited 2015 Aug 19]. Available from: http://globocan.iarc.fr/Default.aspx

[2] Roy S, Mandal TK, Das S, Srinivas S, Agarwal A, Gupta A, Singh A, Singh A, Kuraparthy S, Kapoor A, Ghosh R (2020) Demography and pattern of care of patients with head-and-neck carcinoma: experience from a tertiary care center in North India. Cancer Res Stat Treat 3:730–735

[3] Sankaranarayanan R, Masuyer E, Swaminathan R, Ferlay J, Whelan S (1998) Head and neck cancer: a global perspective on epidemiology and prognosis. Anticancer Res. 18(6B):4779–86 9891557

[4] Pfister DG, Spencer S, Adelstein D, Adkins D, Anzai Y, Brizel DM et al (2020) Head and neck cancers version 2.2020 NCCN Clinical Practice Guidelines in Oncology. J Natl Compr Canc Netw 18(7):873–98 32634781 10.6004/jnccn.2020.0031

[5] Patil VM, Muthuluri H, Choudhary J, Parekh D, Abraham G, Noronha V, Menon N, Dhumal S, Prabhash K (2022) Nivolumab in platinum-refractory head-and-neck cancers: a retrospective observational audit from a tertiary cancer center. Cancer Res Stat Treat. 5:468-73

[6] Rajappa SJ, Pinninti R (2022) Real-world evidence with nivolumab in head-and-neck cancer: access is key!. Cancer Res Stat Treat. 5(3):541-3

[7] Ferris RL, Blumenschein G Jr, Fayette J, Guigay J, Colevas AD, Licitra L, et al. (2016) Nivolumab for recurrent squamous-cell carcinoma of the head and neck. N Engl J Med. 375(19):1856–67 10.1056/NEJMoa1602252 PMC5564292 27718784

[8] Cohen EEW, Soulières D, Le Tourneau C, Dinis J, Licitra L, Ahn M-J, et (2019) al. Pembrolizumab versus methotrexate, docetaxel, or cetuximab for recurrent or metastatic head-and-neck squamous cell carcinoma (KEYNOTE-040): a randomised, open-label phase 3 study. Lancet. 393(10167):156–67 10.1016/S0140-6736(18)31999-8 30509740

[9] Burtness B, Harrington KJ, Greil R, Soulières D, Tahara M, de Castro G Jr, et al. (2019) Pembrolizumab alone or with chemotherapy versus cetuximab with chemotherapy for recurrent or metastatic squamous cell carcinoma of the head and neck (KEYNOTE-048): a randomised, open-label, phase 3 study. Lancet. 394(10212):1915–28 10.1016/S0140-6736(19)32591-7 31679945

[10] Mathur P, Sathishkumar K, Chaturvedi M, Das P, Sudarshan KL, Santhappan S, et al. (2020) Cancer Statistics: report from National Cancer Registry Programme India. JCO Global Oncology [Internet]. [cited 2021 Dec 15]; Available from: 10.1200/GO.20.00122 10.1200/GO.20.00122 PMC7392737 32673076

[11] Patil VM, Noronha V, Joshi A, Agarwal J, Ghosh-Laskar S, Budrukkar A, et al. (2019) A randomized phase 3 trial comparing nimotuzumab plus cisplatin chemoradiotherapy versus cisplatin chemoradiotherapy alone in locally advanced head and neck cancer. Cancer [Internet]. 10.1002/cncr.32179 10.1002/cncr.32179 31150120

[12] Murthy V, Calcuttawala A, Chadha K, d’Cruz A, Krishnamurthy A, Mallick I, et al. (2017) Human papillomavirus in head and neck cancer in India: current status and consensus recommendations. South Asian J Cancer. 6(3):93–8 10.4103/sajc.sajc_96_17 PMC5615888 28975111

[13] Nandi S, Mandal A, Chhebbi M (2021) The prevalence and clinicopathological correlation of human papillomavirus in head and neck squamous cell carcinoma in India: a systematic review article. Cancer Treat Res Commun 26:100301 33401132 10.1016/j.ctarc.2020.100301

[14] Gillison ML, Chaturvedi AK, Anderson WF, Fakhry C (2015) Epidemiology of human papillomavirus-positive head and neck squamous cell carcinoma. J Clin Oncol. 33(29):3235–42 10.1200/JCO.2015.61.6995 PMC4979086 26351338

[15] Lyu X, Zhang M, Li G, Jiang Y, Qiao Q (2019) PD-1 and PD-L1 expression predicts radiosensitivity and clinical outcomes in head and neck cancer and is associated with HPV infection. J Cancer. 10(4):937–48 10.7150/jca.27199 PMC6400795 30854100

[16] Uppaluri R, Zolkind P, Lin T, Nussenbaum B, Jackson RS, Rich J et al. (2017) Neoadjuvant pembrolizumab in surgically resectable, locally advanced HPV negative head and neck squamous cell carcinoma (HNSCC). J Clin Orthod. 35(15_suppl):6012–6012

[17] Schoenfeld JD, Hanna GJ, Jo VY, Rawal B, Chen Y-H, Catalano PS et al. (2020) Neoadjuvant nivolumab or nivolumab plus ipilimumab in untreated oral cavity squamous cell carcinoma: a phase 2 open-label randomized clinical trial. JAMA Oncol. 6(10):1563–70 10.1001/jamaoncol.2020.2955 PMC7453348 32852531

[18] Uppaluri R, Campbell KM, Egloff AM, Zolkind P, Skidmore ZL, Nussenbaum B et al (2020) Neoadjuvant and adjuvant pembrolizumab in resectable locally advanced human papillomavirus-unrelated head and neck cancer: a multicenter phase II trial. Clin Cancer Res 26(19):5140–5152 32665297 10.1158/1078-0432.CCR-20-1695 PMC7547532

[19] D’Angelo SP, Shoushtari AN, Agaram NP, Kuk D, Qin L-X, Carvajal RD et al (2015) Prevalence of tumor-infiltrating lymphocytes and PD-L1 expression in the soft tissue sarcoma microenvironment. Hum Pathol 46(3):357–365 25540867 10.1016/j.humpath.2014.11.001 PMC5505649

[20] Chow LQM, Haddad R, Gupta S et al (2020) Antitumor activity of pembrolizumab in biomarker-unselected patients with recurrent and/or metastatic head and neck squamous cell carcinoma: results from the phase Ib KEYNOTE-012 expansion cohort. J Clin Oncol 34(32):3838–3845. 10.1200/JCO.2016.68.1478 10.1200/JCO.2016.68.1478 PMC6804896 27646946

[21] Bell A, Taylor C, Bannerjee T et al (2018) Comparison of PD-L1 expression in matched biopsy and resection specimens of non-small cell lung cancer. Histopathology 72(7):1117–1126. 10.1111/his.13505

[22] Rimm DL, Han G, Taube JM et al (2021) A prospective multi-institutional, pathologist-based assessment of 4 immunohistochemistry assays for PD-L1 expression in non–small cell lung cancer. JAMA Oncol 3(8):1051–1058. 10.1001/jamaoncol.2017.0013 10.1001/jamaoncol.2017.0013 PMC5650234 28278348

[23] Ferris RL, Blumenschein G Jr, Fayette J, et al. (2016) Nivolumab for recurrent squamous-cell carcinoma of the head and neck. N Engl J Med. 375(19):1856-1867. 10.1056/NEJMoa1602252 10.1056/NEJMoa1602252 PMC5564292 27718784

[24] Kim HR, Ha SJ, Hong MH et al (2019) PD-L1 expression on tumor-infiltrating immune cells as a predictive biomarker for the efficacy of immune checkpoint inhibitors in non-small cell lung cancer. Sci Rep 9(1):1–9. 10.1038/s41598-019-39607-330626917

[25] Cerbelli B, Girolami I, Eccher A, Costarelli L, Taccogna S, Scialpi R, et al. (2021) Evaluating programmed death-ligand 1 (PD-L1) in head and neck squamous cell carcinoma: concordance between the 22C3 PharmDx assay and the SP263 assay on whole sections from a multicentre study. Histopathology [Internet]. 10.1111/his.14562 10.1111/his.14562 PMC9299113 34496080

